# Study on the factors influencing the impaired abilities of daily living in middle-aged and older adult arthritis patients based on binary logistic regression and categorical decision tree model

**DOI:** 10.3389/fpubh.2025.1531872

**Published:** 2025-06-03

**Authors:** Bao-xuan Zhang, Jin-ping Luo, Jia-ying Sun, Ming-hui Geng, Yi-fan Mou, Nan-nan Cheng, Zhao-xuan Wang, Wen-qiang Yin, Zhong-ming Chen, Dong-ping Ma

**Affiliations:** ^1^School of Management, Shandong Second Medical University, Shandong, China; ^2^"Health Shandong"Severe Social Risk Prevention and Management Synergy Innovation Center, Shandong, China; ^3^Collaborative Innovation Center of Social Risks Governance in Health, Shanghai, China

**Keywords:** activities of daily living, arthritic, middle-aged and older adults, pain, CHARLS

## Abstract

**Background:**

Arthritis is the most disabling disease worldwide, and the presence of the disease usually greatly threatens the patient’s activities of daily living (ADL). Currently, there are a few studies that are related to exploring factors associated with impaired ADL in middle-aged and older adult arthritis patients. This study aimed to explore the factors associated with impaired ADL in Chinese middle-aged and older adult patients through logistic regression and decision tree models.

**Methods:**

The method of univariate analysis was the chi-square test. Variables with significant differences in univariate analysis were included in binary logistic regression model and decision tree model based on the E-CHAID algorithm to explore the factors associated with impaired ADL in middle-aged and older adult arthritis patients in China.

**Results:**

The results of the logistic regression model indicated that sex, place of residence, age, education level, falls, Internet usage, depressive symptoms, pain, self-rated health, and number of comorbid chronic diseases were the influencing factors for impaired ADL. The decision tree results showed that pain was the most important variable predicting impaired ADL in middle-aged and older adult arthritis patients. The area under the curve of the logistic regression model and the decision tree model were 0.792 (95%CI: 0.780–0.804) and 0.767 (95%CI: 0.754–0.780), respectively.

**Conclusion:**

The results of the study suggest that pain, self-rated health, Internet usage, age, and depressive symptoms are significant correlates of impaired ADL. Primary care providers need to provide intervention strategies that are individualized to the middle-aged and older adults with arthritis themselves.

## 1 Introduction

Arthritis is a chronic inflammatory disease with a high global prevalence (1), and patients usually bear the burden of disease, which negatively affects their quality of life (2, 3). In 2015, a study showed that prevalence of arthritis in middle-aged and older adults had reached 31.37% in China, and it was confirmed that the prevalence increased with age (4). In addition, during 2013 to 2015, the prevalence of arthritis in American adults has reached 22.7% (5). And a study suggested that the number of people with rheumatoid arthritis has reached 17.6 million worldwide in 2020, and predicted that the number of people with the disease will continue to increase by 2050 (6), which showed that the prevalence of rheumatoid arthritis is a serious situation.

Patients with arthritis often have impaired mobility and limited ability to perform activities of daily living (ADL). In 2008, a study with a 10-year follow-up found that arthritis significantly increased the risk of losing the ability to live an independent life, and that middle-aged patients were more likely to experience mobility and ADL difficulties as they entered old age (7). In 2021, a study showed that the number of arthritis-attributable activity limitations accounted for 43.9% of patients with arthritis in the United States, during from 2016 to 2018, with a linear increase over the past 20 years (8). Similarly, the prevalence of disability in ADL in China has been increasing from 2011 to 2018 (9), and the risk of ADL impaired in middle-aged and older adults with arthritis was 38% higher compared to those without arthritis (10), which showed that ADL in middle-aged and older adults with arthritis in China need to be focused on. It has been shown that impaired ability to perform basic activities of daily living (BADL) reducing subjective well-being by decreasing the likelihood of engaging in leisure activities (11), and impaired ability to perform ADL increasing the risk of suicide and even death (12), which greatly affects the quality of life of patients. Therefore, understanding the influencing factors of impaired ADL is important for maintaining the multidimensional health of middle-aged and older adult arthritis patients and promoting active aging.

In recent years, the perspective of many studies has been related to impaired ADL. For example, logistic regression has been used to find that sex, age, and chronic disease are influential factors in impaired ability to perform ADL (13, 14). Gower et al. found that the structure of pain mediated the effect between pain intensity and pain interference and ADL through the use of SPSS Hayes (15). Demir et al. found that that cognitive abilities may contribute to the deterioration of ADL and independent functioning in older adult adults (16). Nguyen et al. used longitudinal data to demonstrate that depressive symptoms is a correlate of functional limitations (17). Although there are studies focusing on the influencing factors of impaired ADL, there is a lack of research results for Chinese middle-aged and older adult arthritis patients, and there are a few studies comparing the predictive effects using the two methods. Decision tree model, as a machine learning algorithm with logical criteria, is able to reveal unspecified interrelationships among factors through tree diagrams, which better compensates for the limitation of regression analysis that can only test *a priori* specified interaction effects (18–20). Consequently, the current study was designed to focus on Chinese middle-aged and older adults aged 45 years and above, and to explore the main influencing factors of arthritis patients’ impaired ADL by using logistic regression model and categorical decision tree model, in order to provide theoretical basis and intervention strategies for the improvement of arthritis patients’ ability to perform daily activities in the middle-aged and older adults.

## 2 Methods

### 2.1 Data sources

The data for this study came from the 2020 China Health and Retirement Longitudinal Study (CHARLS), a representative survey of people aged 45 and older in mainland China, its national baseline survey was conducted in 2011, using multi-stage sampling and proportional population sampling techniques, which covers 150 districts and counties in 28 provinces in China, which encompasses multiple dimensions of personal health and functioning (21, 22), and is able to provide a more comprehensive source of data for this study.

For the current study, we selected data from the 2020 CHARLS survey, which had a total of 19,395 participants. First, because there were 19,367 participants in the “Health Status and Functioning” section, we included a total of 19,367 participants after combining this section, and then we excluded 238 participants under 45 years of age and 12,644 participants 45 years of age and older who did not have arthritis, retaining 6,485 participants. Subsequently, we excluded participants with abnormal or missing data on cognitive functioning (*n* = 701), depressive symptoms (*n* = 532), and self-rated health status (*n* = 1). For example, failing to take the assessment (respondents were unable to complete it for objective physical reasons) or refusing to answer the questions. We ultimately included a total of 5,251 participants.

### 2.2 Assessment of ADL

The ability to perform ADL was measured by the Basic Activities of Daily Living (BADL) scale and the Instrumental Activities of Daily Living (IADL) scale (23). BADL includes six items: dressing, bathing, eating, getting in and out of bed, going to the toilet, and controlling urination and defecation; and IADL includes six items: doing chores, cooking, shopping, making phone calls, taking medication, and managing money. According to the options “no difficulty,” “difficult but still can be completed,” “difficult, need help,” “cannot be completed” were assigned values of 1, 2, 3, and 4, separately, and the total score ranged from 12 to 48 points. In this study, a total score of 12 was defined as unimpaired ADL, and a score higher than 12 was classified as impaired ADL (24). Shen et al. explained that ADL scale has strong internal consistency and good retest reliability in their article (25). In the current sample, the internal consistency coefficient (Cronbach’s *α*) of the ADL scale was 0.838.

### 2.3 Assessment of depressive symptoms

In CHARLS, depressive symptoms were measured by the Center for Epidemiologic Studies Depression Short Form (CESD-10), which consists of 10 questions, two of which are positive items, “I am hopeful about the future” and “I am happy.” In the negative items, subjects answered “little or nothing,” “not too much,” “sometimes or half the time,” and “most of the time” were assigned scores of 0, 1, 2, and 3, respectively. On the contrary, in the positive items, subjects who answered “little or nothing,” “not too much,” “sometimes or half the time,” and “most of the time” were assigned scores of 3, 2, 1, and 0, respectively, and a total score ranging from 0 to 30, with a score of 10 and above was recognized as the presence of depressive symptoms (26, 27), where a score of less than 21 was defined as mild–moderate depressive symptoms, and a score of 21 and above was defined as severe depressive symptoms (28). The CESD-10 scale has good reliability and validity in the application of measuring depressive symptoms in Chinese middle-aged and older adult people (29). In this study, the internal consistency coefficient (Cronbach’s *α*) of the CESD-10 was 0.797.

### 2.4 Assessment of cognitive impairment

According to previous studies, cognitive impairment was measured by the Mental State Examination (MMSE) Scale. It includes four dimensions: orientation, numeracy, drawing ability, and situational memory. Orientation was measured by asking about the season, year, month, day, and day of the week, and is scored out of 5, with 1 point for each correct answer; numeracy was measured by asking the respondent to subtract 7 from 100 consecutively (a total of 5 times), and is scored out of 5, with 1 point for each correct answer; drawing ability was determined by observing the correctness of the respondent’s drawing, with a full score of 1. Situational memory included both instantaneous and delayed memory, and which scores range from 0 to 20 points. The total score of the Cognitive Impairment Scale ranges from 0 to 31 points, and in this study, In this study, a total score of less than 11 was classified as having cognitive impairment (30, 31). The Cronbach’s *α* for the MMSE scale in this study was 0.818.

### 2.5 Assessment of other variables

Other variables included basic demographic characteristics and health-related information. Specifically, basic demographic characteristics included sex (male and female), place of residence (urban and rural), marital status (married and unmarried), age (45–60 years and ≥60 years), education level (elementary school and lower, junior high school, high school and higher), pension insurance (yes and no). Health-related information included medical insurance (yes and no), falls (yes and no), whether or not you are in contact with or see your child on a weekly basis (yes and no), Internet usage (yes and no), pain (none, mild to moderate pain, severe pain), self-rated health (good, general, not good), number of other comorbid chronic conditions (0, 1–2, 3), and participation in social activities (yes and no).

Marital status was categorized as married or unmarried, if the answer was “separated (no longer living together as spouses)” or “divorced” or “widowed” or “never married,” the marital status was defined as “unmarried.”

Falls were categorized as yes or no. Falls were defined as “Yes” if the respondent answered “Yes” to the question “Have you had any falls since the last visit.”

Self-rated health is measured by a question in the questionnaire, which is “How do you think your health is?.” In this study, self-rated health was categorized as good, general, or poor, if participants gave answers of “good” and “very good,” they were considered for inclusion into the good population, and if respondents answered “not good,” “very bad,” they were defined as not good (32).

The questionnaire’s question, “How often do you suffer from pain?” was used as a measure of pain for this study, if respondents answered “not at all,” “a little or some,” “more or very much,” pain was defined as none, mild to moderate pain, and severe pain.

### 2.6 Statistical analysis

In this study, we used SPSS 27 for data analysis and processing, describing the basic demographic characteristics of the study population in terms of frequencies and percentages, and verifying the differences between the ADL-impaired and ADL-unimpaired groups in different middle-aged and older adult arthritis patients by using the chi-square test. Subsequently, we incorporated statistically significant variables from the chi-square test into a binary logistic regression analysis model and a categorical decision tree model to identify the main correlates of impaired ADL (20). Prior to the regression analysis, we tested for the presence of multicollinearity among the variables by means of a multicollinearity diagnostic. The binary logistic regression analysis (forward: LR) was used to to identify factors influencing impaired ADL. The categorical decision tree model utilized the exhaustive chi-square automatic interaction detection (E-CHAID) (33) growth method, which maximum tree depth was set to three, with a minimum sample size of 100 for the parent nodes and 50 for the child nodes, and tree pruned by 10-fold cross validation method. Finally, receiver operating characteristic (ROC) curves were plotted, and the predictive ability of the two models was assessed by calculating the area under curve (AUC). In this study, the two-sided test level *α* = 0.05.

The dependent variable in this study was ADL. Independent variables included sex, place of residence, marital status, marital status, age, education level, pension insurance, medical insurance, falls, whether or not you are in contact/see your child on a weekly basis, internet usage, depressive symptoms, cognitive. Impairment, pain, self-rated health, number of comorbid chronic diseases and participation in social activities. Furthermore, to ensure that the results of the logistic regression model and the decision tree model are easier to interpret, we assigned the variables at the same level (Table 1).

**Table 1:** Description of variable assignment.

Variable	Assignment
Dependent variable	
ADL impaired	No = 1; Yes = 2
Independent variable	
Sex	Male = 1, Female = 2
Place of residence	Urban = 1, Rural = 2
Marital status	Married = 1; unmarried = 2
Age	<60 = 1; ≥ 60 = 2
Education level	Elementary school and lower = 1; Junior high school = 2; High school and higher = 3
Pension insurance	No = 1; Yes = 2
Medical insurance	No = 1; Yes = 2
Falls	No = 1; Yes = 2
Whether or not you are in contact/see your child on a weekly basis	No = 1; Yes = 2
Internet usage	No = 1; Yes = 2
Depressive symptoms	None = 1; Mild to moderate depressive symptoms = 2; Major depressive symptoms = 3
Cognitive impairment	No = 1; Yes = 2
Pain	None = 1; Mild to moderate pain = 1; Severe pain = 1
Self-rated health	Good = 1; General = 2; Not good = 3
Number of comorbid chronic diseases	0 = 1; 1–2 = 2; ≥ 3 = 3
Participation in social activities	No = 1; Yes = 2

## 3 Results

### 3.1 Basic information about the research subjects

This study included 5,251 patients with arthritis, 2,195 (41.80%) males and 3,056 (58.20%) females. There were 1,636 (31.16%) urban residents and 3,615 (68.84%) rural residents. There were 4,387 (83.55%) married residents, 2,128 (40.53%) residents aged <60 years, and 1,099 (20.93%) with junior high school education. The number of people with impaired ADL was 2,255 (42.94%). The results of the chi-square test showed that there were significant differences in different ADL groups in the following areas (*P* < 0.05), including sex, place of residence, marital status, age, education level, medical insurance, falls, weekly meetings or contact with children, internet usage, depressive symptoms, cognitive impairment, pain, self-rated health, number of comorbid chronic diseases, and participation in social activities (Table 2).

**Table 2:** Basic profile of the study population (*n* = 5,251).

Variable	Category	*n*(%)	ADL impaired		χ^2^	*P*
			Yes	No		
Sex	Male	2,195 (41.80)	775	1,420	89.769	<0.001
	Female	3,056 (58.20)	1,480	1,576		
Place of residence	urban	1,636 (31.16)	572	1,064	61.775	0.002
	Rural	3,615 (68.84)	1,683	1,932		
Marital status	Married	4,387 (83.55)	1,790	2,597	49.919	<0.001
	Unmarried	864 (16.45)	465	399		
Age	<60	2,128 (40.53)	694	1,434	155.868	<0.001
	≥60	3,123 (59.47)	1,561	1,562		
Education level	Elementary school and lower	3,687 (70.22)	1,776	1,911	146.643	<0.001
	Junior high school	1,099 (20.93)	363	736		
	High school and higher	465 (8.86)	116	349		
Pension insurance	Yes	4,554 (86.73)	1,962	2,592	0.270	0.603
	No	697 (13.27)	293	404		
Medical insurance	Yes	5,032 (95.83)	2,139	2,893	9.371	0.002
	No	219 (4.17)	116	103		
Falls	Yes	1,153 (21.96)	710	443	209.373	<0.001
	No	4,098 (78.04)	1,545	2,553		
Weekly meetings or contact with children	Yes	4,222 (80.40)	1,754	2,468	17.232	<0.001
	No	1,029 (19.60)	501	528		
Internet usage	Yes	1,948 (37.10)	621	1,327	154.756	<0.001
	No	3,303 (62.90)	1,634	1,669		
Depressive symptoms	None	2,634 (50.16)	731	1,903	577.700	<0.001
	Mild to moderate depressive symptoms	2,108 (40.14)	1,138	970		
	Major depressive symptoms	509 (9.69)	386	123		
Cognitive impairment	Yes	1,359 (25.88)	734	625	91.638	<0.001
	No	3,892 (74.12)	1,521	2,371		
Pain	None	1,238 (23.58)	266	972	676.535	<0.001
	Mild to moderate pain	2,455 (46.75)	922	1,533		
	Severe pain	1,558 (29.67)	1,067	491		
Self-rated health	Good	756 (14.40)	145	611	614.309	<0.001
	General	2,684 (51.11)	930	1,754		
	Not good	1,811 (34.49)	1,180	631		
Number of comorbid chronic diseases	0	1,032 (19.65)	325	707	155.594	<0.001
	1 ~ 2	2,497 (47.55)	995	1,502		
	≥3	1,722 (32.79)	935	787		
Participation in social activities	Yes	2,572 (48.98)	1,057	1,515	7.025	0.008
	No	2,679 (51.02)	1,198	1,481		

### 3.2 Colinearity diagnosis

The diagnosis of multicollinearity was applied to determine the independence of the factors in order to ensure the accuracy of the estimates of the regression models. In this study, the variables which were statistically meaningful in the univariate analysis were used as independent variables for the diagnosis of multicollinearity, and the analysis results indicated that the tolerance range of the 15 independent variables included was from 0.711 to 0.980, and the range of Variance Inflation Factors (VIF) was from 1.021 to 1.407, so this study concluded that there was no multicollinearity among the variables (34) (Table 3).

**Table 3:** Diagnosis of multicollinearity.

Variable	Collinearity diagnostics		Variable	Collinearity diagnostics	
	Tolerance	VIF		Tolerance	VIF
Sex	0.901	1.110	Internet usage	0.711	1.407
Place of residence	0.880	1.137	depressive symptoms	0.786	1.272
Marital status	0.909	1.110	Cognitive impairment	0.838	1.194
Age	0.797	1.254	Pain	0.757	1.321
Education level	0.788	1.268	Self-rated health	0.767	1.304
Medical insurance	0.976	1.024	Number of comorbid chronic diseases	0.896	1.116
Falls	0.941	1.063	Participation in social activities	0.960	1.042
Weekly meetings or contact with children	0.980	1.021			

### 3.3 Binary logistic regression analysis

Binary logistic regression models indicated that sex, place of residence, age, education level, falls, Internet usage, depressive symptoms, pain, self-rated health, and number of comorbid chronic diseases were influential factors for impaired ADL (*P* < 0.05). Specifically, compared to the reference category, females (OR = 1.359, 95% CI: 1.189–1.554), rural (OR = 1.213, 95% CI: 1.048–1.404), 60 years and older (OR = 1.882, 95% CI: 1.630–2.175), and history of falls (OR = 1.743, 95% CI. 1.494–2.034), mild to moderate depressive symptoms (OR = 1.878, 95%CI: 1.640–2.151), major depressive symptoms (OR = 3.151, 95%CI: 2.465–4.029), mild to moderate pain (OR = 1.640, 95%CI: 1.379–1.951), severe pain (OR = 3.404, 95%CI: 2.794–4.148), self-rated health as general (OR = 1.557, 95%CI: 1.255–1.932), self-rated health as not good (OR = 3.306, 95%CI: 2.618–4.174), and the number of co-morbid chronic diseases 3 or more (OR = 1.360, 95%CI: 1.124–1.644) were risk factors for impaired ADL. High school education and above (OR = 0.649, 95%CI: 0.499–0.844), and internet usage (OR = 0.724, 95%CI: 0.622–0.842) were protective factors for impaired ADL (Table 4).

**Table 4:** Results of binary logistic regression analysis.

Variables	β	SE	Wald	*P*	OR	95%CI
Sex	0.307	0.068	20.215	<0.001	1.359	1.189–1.554
Place of residence	0.193	0.075	6.677	0.010	1.213	1.048–1.404
Age	0.633	0.074	73.917	<0.001	1.882	1.630–2.175
Education level (Ref: Elementary school and lower)			10.722	0.005		
junior high school	−0.109	0.087	1.573	0.210	0.897	0.757–1.063
High school and higher	−0.432	0.134	10.386	0.001	0.649	0.499–0.844
Falls	0.556	0.079	49.965	<0.001	1.743	1.494–2.034
Internet usage	−0.323	0.077	17.485	<0.001	0.724	0.622–0.842
depressive symptoms (Ref: None)			126.995	<0.001		
Mild to moderate depressive symptoms	0.630	0.069	82.744	<0.001	1.878	1.640–2.151
Major depressive symptoms	1.148	0.125	83.795	<0.001	3.151	2.465–4.029
Pain (Ref: None)			160.064	<0.001		
Mild to moderate pain	0.495	0.089	31.181	<0.001	1.640	1.379–1.951
Severe pain	1.225	0.101	147.694	<0.001	3.404	2.794–4.148
Self-rated health (Ref: Good)			145.907	<0.001		
General	0.443	0.110	16.152	<0.001	1.557	1.255–1.932
Not good	1.196	0.119	100.923	<0.001	3.306	2.618–4.174
Number of comorbid chronic diseases (Ref:0)			11.761	0.003		
1–2	0.110	0.090	1.498	0.221	1.116	0.936–1.330
≥3	0.307	0.097	10.031	0.002	1.360	1.124–1.644

### 3.4 Categorical decision tree analysis

**Figure 1:**
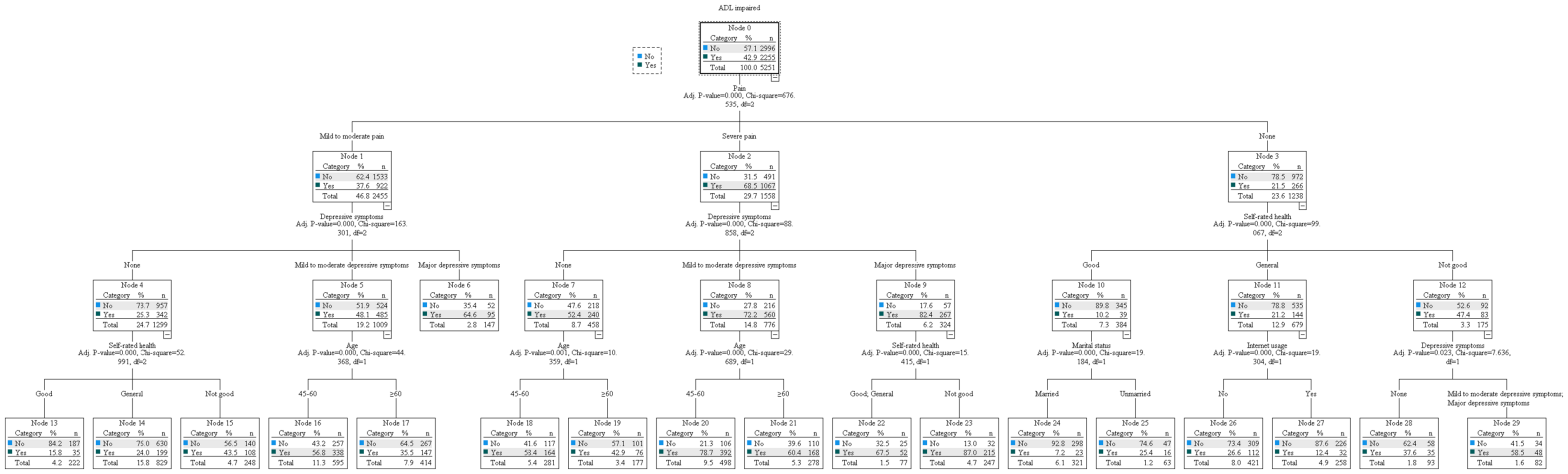
Tree diagram of categorical decision tree.

The results of the categorical decision tree modeling showed that impaired ADL were mainly associated with pain, depressive symptoms, self-rated health, age, marital status, and Internet use. The variable at the first level of the tree structure was pain, with a higher detection rate of impaired ADL in patients with severe pain (68.5%) than in patients with mild-to-moderate pain (37.6%) and none (21.5%). The variables in the second level of the tree were depressive symptoms and self-rated health, with patients suffering from major depressive symptoms having a higher detection rate of impaired ADL in the group of patients with severe pain (82.4%) or mild to moderate pain (64.6%), and a higher rate of impaired ADL in the group with not good self-rated health (47.4%) in the group of patients with none pain. A total of five variables were screened in the third level of the tree, including self-rated health, depressive symptoms, age, Internet usage, and marital status, with a higher rate of impaired ADLs (87.0%) in patients with severe pain, severe depressive symptoms, and poor self-rated health (Figure 1).

### 3.5 Comparison of the results of the two models

**Figure 2:**
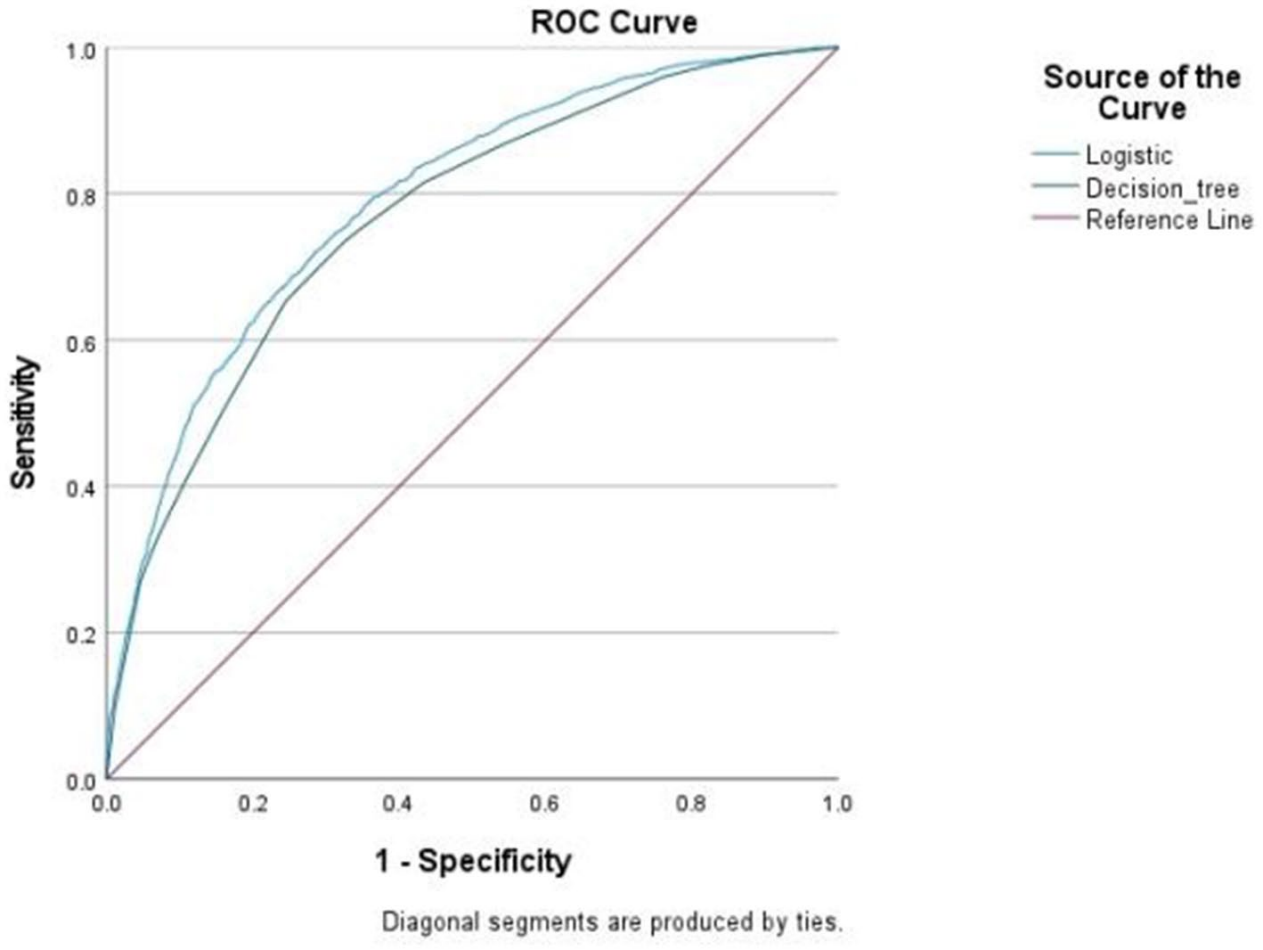
Binary logistic vs. categorical decision tree ROC curves.

The results of both models showed that age, self-rated health, Internet usage, pain, and depressive symptoms were influential factors in the impaired ADL in middle-aged and older adult arthritis patients. ROC curves were plotted based on the predictive probabilities obtained from both models as test variables. The ROC curves of the two models were far from the diagonal, indicating that the models used in this study had some predictive effect. But it is worth noting that the categorical decision tree model eliminated the five statistically significant influencing factors, namely, sex, place of residence, education level falls, and number of comorbidities with other chronic diseases in the binary logistic regression, while marital status in the categorical decision tree was not statistically significant in the binary logistic. In addition, the area under the ROC curve of the dichotomous logistic regression model was 0.792 (95% CI: 0.780–0.804; *P* < 0.001), with a sensitivity of 70.47% and a specificity of 73.79%, while the area under the ROC curve of the categorical decision tree model was 0.767 (95% CI: 0.754–0.780), with a sensitivity of 66.75% and a specificity of 74.29%. The predictive effectiveness of the two models was moderate (0.7–0.9), and the categorical decision tree model was slightly lower than the binary logistic regression model (Figure 2).

## 4 Discussion

In this study, the impaired ADL rate of Chinese middle-aged and older adult arthritis patients was 42.94%. It was significantly higher than the finding of Tian et al., who found that the impaired ADL rate of middle-aged and older adult Chinese had reached 15.23% from 2011 to 2018 (35). It was also higher than the finding of Gao et al. who found that the impaired ADL rate of Chinese older adult was 26.56%. This phenomenon can be attributed to the pathogenesis and disease symptoms of arthritis itself. Patients with arthritis are highly likely to have damage to their joints (13). Yano et al. found that foot and ankle problems in patients with arthritis, such as symptoms of swelling, stiffness, and structural deformities, often lead to higher disease activity and higher functional disability (36). In addition, the prevalence of pain and fatigue, as well as the unpredictability of symptoms, contributes largely to the frustration and isolation felt by arthritis patients, making their ADL limited and leading to an increase in sedentary time (37).

This study found that binary logistic regression models yielded different influences than categorical decision tree models. For example, the categorical decision number model did not indicate that gender, place of residence, falls, or number of comorbid chronic conditions could influence impaired ADL. It is possible that this was limited by the depth of the decision tree and the sample size at each node, or that the effects of these variables were relatively weak and were pruned out during the growth of the tree model.

### 4.1 Effect of age on ADL

This study showed that the risk of impaired ADL in the 60 and older group was 1.359 times higher than that in the under60 group, and that the risk of impaired ADL in arthritis patients increases with age. This is similar to the findings of Hu et al. who confirmed the increase in impaired ADL with age through an age-period-cohort cross-classified random effects model (38). Similarly, the findings of Chen et al. showed a strong relationship between disability rates and age (39). In the case of arthritic patients, it may be due to the presence of cartilage damage and loosening of supporting ligaments in arthritic patients (40), Ai et al. also mentioned in their study that the decline in physical function led to further damage to their inflammatory areas, leading to an inability to exercise with force thus making them more susceptible to impaired ADL (10).

### 4.2 Pain and ADL

Pain is usually the more obvious symptom of arthritis, and the intrinsic mechanism of pain may be that arthritis affects the central nervous system, which acts in conjunction with peripheral inflammatory changes to cause central sensitization, resulting in chronic pain in the body (41, 42). In this study, pain was found to be an important risk factor for impaired ADL in middle-aged and older adult patients with arthritis, the risk of impaired ADLs was 3.404 times higher in the severe pain group and 1.640 times higher in the mild–moderate pain group compared to the none group. This is consistent with the findings of James et al. (43). Muhammad et al. found a significant association between the progression of arthritis pain and increased disability, with patients with persistently worsening pain or chronic severe pain having more pronounced functional impairment relative to respondents whose pain was mild and remained stable (44). This may be due to frequent pain leading to pain catastrophizing, which in turn amplifies the patient’s fear and leads to a vicious cycle of individual participation restriction. Meanwhile, fear avoidance beliefs play an important role in predicting physical activity levels. For example, Larsson et al. found that pain patients with low kinesiophobia possessed higher levels of physical activity (OR = 0.95, 95% CI: 0.91–0.99) (45). In addition, activity-modifying behaviors have a mediating effect between pain and physical functioning and may counteract the effects of joint pain on physical functioning (46). Therefore, people with arthritis need to adjust their activity behaviors in a timely manner based on pain to avoid damage to their ADL. Healthcare providers need to develop mechanisms to monitor the dynamics of arthritis pain, be sensitive to the pain experience of people with arthritis, and manage the pain of people with arthritis through the provision of individualized, patient-centered services (47).

### 4.3 Depressive symptoms and ADL

The negative role of depressive symptoms in the health of patients with arthritis cannot be ignored. The study by Ke et al. demonstrated a bidirectional association between depressive symptoms and arthritis, with the two acting as risk factors for each other and forming an underlying pathologic cycle (48). Depressive symptoms is also a key mediating variable linking arthritis to all-cause mortality and cardiovascular disease mortality (49). Of note, compared to the none group, the present study found that the risk of impaired ADL was increased 3.151 times in the major depression symptoms group and 1.878 times in the mild–moderate depression symptoms group, depressive symptoms was a risk factor for imparied ADL in middle-aged and older adult with arthritis, which is consistent with previous findings. Carrière et al. found that depressive symptoms was an independent predictor of disability in the older adult population and increased the risk of impaired ADL as the level of depressive symptoms worsened (50). Zhou et al. found that, compared to middle-aged and older adult without depressive symptoms, the rate of ADL disability was higher in the depressed population (23). Depressive symptoms as a common psychological state, the level of depressive symptoms will affect the patient’s state of perceived stress (51), which will easily cause individuals to produce a positive or negative evaluation of subjective and objective things, thus indirectly affecting their own judgment of ADL. At the same time, depressive symptoms not only significantly reduce an individual’s level of motivation to participate in leisure activities, but more importantly, this diminished interest increases the negative impact of depressive symptoms on physical functioning by weakening the frequency and intensity of participation in leisure activities (52). Additionally, people who eat a healthy diet typically have a lower risk of ADL restriction (53), and depressive symptoms may contribute to the phenomenon of loss of appetite in patients, leading to malnutrition in the body (54), which is more likely to result in functional impairment. In order to reduce the detrimental effects of depressive symptoms on ADL in patients with arthritis, general practitioners need to collaborate with community resources to conduct regular routine depressive symptoms screening, build bridges of communication with patients, and strengthen family support for patients.

### 4.4 The internet and ADL

Our study found that Internet usage was a protective factor for impaired ADL. This is consistent with the findings of Wen et al. who found that Internet usage had a positive impact on the health status of middle-aged and older adult, with individuals who used the Internet being less likely to have impaired ADL compared to individuals who did not use the Internet (OR = 0.48, 95% CI: 0.39-0.60) (55). Similarly, Ding et al. demonstrated that Internet usage reduced imparied ADL by 1.1 percentage points (56). This may be due to the fact that Internet usage allows middle-aged and older adult to be exposed to health-related information, such as diet, nursing care, and exercise, which can be utilized in health care (57). At the same time, the Internet can break the geographical limitation to communicate with family members or friends, and family members can better understand their health status and intervene in a timely manner. Similarly, perceptions of the perceived importance of the Internet have a significant positive impact on family climate and behavioral independence, and can improve life satisfaction and self-rated health to some extent (58). Therefore, communities with middle-aged and older adult with arthritis need to popularize basic knowledge and usage of the Internet, emphasize the importance of Internet usage, and enhance digital skills training.

### 4.5 Self-rated health and ADL

Self-rated health is the evaluation of one’s own health based on subjective feelings, and this study found that the poorer the self-rated health, the more impaired the ability to perform daily activities in middle-aged and older adult arthritis patients. Fujiwara et al. found that self-rated health was a good predictor of improving and preventing the decline of IADL (59). Zhao et al. found that self-rated health affects the trajectory of physical function, and that people with less than optimal self-rated health usually show a downward trend in physical function over a certain period of time (60). In addition, socioeconomic status, lifestyle, and psychosocial factors are associated with self-rated health. Different socioeconomic statusages have different lifestyles; for example, groups with high socioeconomic statusages may be more inclined to fitness activities. And people with low socioeconomic status may have different self-rated health status compared to those with high socioeconomic status who are unable to satisfy their own medical and health care needs due to financial problems (61). Therefore, primary health care providers should educate arthritis patients about the need to seek medical attention when they have problems and to maintain a positive mindset, so as to improve the self-rated health of middle-aged and older adult patients by taking into account a variety of factors.

### 4.6 Strengths and weaknesses

The advantage of this study is that the tree diagram formed by building a categorical decision tree model can more intuitively show the pathways of impaired ADL in arthritis patients, clearly indicate the interactions between variables and the risk factors associated with impaired ADL in each subgroup, and find the most influential combination of factors on ADL by combining with binary logistic regression. However, it is undeniable that this study only used one period of data from the CHARLS database, which could not confirm the causal relationship between the variables, and will consider exploring the causal order based on multiple periods of data in the future. Meanwhile, this study did not include all factors that may be associated with impaired ability to perform daily activities, for example, targeted physical activity programs or specific types of physical exercise may have an important role in ADL in patients with arthritis. Therefore, additional research through field studies by our own team will be considered in the future. In addition, this study was conducted on middle-aged and older adult patients with arthritis, and the generalization of the findings to the whole population is limited.

## 5 Conclusion

This study combined logistic regression modeling and decision tree modeling to identify pain, self-rated health, depressive symptoms, age, and Internet use as key influences on impaired ADL, which provides an important perspective and scientific basis for improving ADL in middle-aged and older adult arthritis patients in China. Primary healthcare providers should prioritize pain-focused individualized interventions, targeted pain management through pharmacological and non-pharmacological interventions, regular routine depressive symptoms screening to track the mental health status of arthritis patients, attention to digital literacy training for middle-aged and older adult arthritis populations to optimize the use of e-health resources, and the use of Internet platforms to understand arthritis patient’s disease status. At the same time, age-appropriate rehabilitation programs should be implemented for different age groups to improve their ADL and quality of life.

## Data Availability

Publicly available datasets were analyzed in this study. This data can be found at: https://charls.pku.edu.cn.
